# Clinical Manifestations, Risk Factors, and Disease Burden of Rickettsiosis, Cambodia, 2007–2020

**DOI:** 10.3201/eid3106.241752

**Published:** 2025-06

**Authors:** Gerard C. Kelly, Agus Rachmat, Long Khanh Tran, Chonthida Supaprom, Hip Phireak, Satharath Prom, Heng Sopheab, Nora Cleary, Michael von Fricken, Christina M. Farris, Andrew G. Letizia

**Affiliations:** Culmen International, LLC, Alexandria, Virginia, USA (G.C. Kelly, L.K. Tran); AC Investment Co, contractor for NAMRU INDO PACIFIC, Phnom Penh, Cambodia (A. Rachmat, C. Supaprom, H. Phireak); Oxford University Clinical Research Unit, Jakarta, Indonesia (A. Rachmat); Cambodia Ministry of National Defense Department of Health, Phnom Penh (S. Prom); Cambodia National Institute of Public Health, Phnom Penh (H. Sopheab); University of Florida College of Public Health and Health Professions, Gainesville, Florida, USA (N. Cleary, M. von Fricken); US Naval Medical Research Command Viral and Rickettsial Diseases Department, Silver Spring, Maryland, USA (C.M Farris); US Naval Medical Research Unit INDO PACIFIC, Singapore (A.G. Letizia)

**Keywords:** rickettsioses, rickettsia infections, arthropods, epidemiology, Mekong Region, vector-borne infections, bacteria, Cambodia

## Abstract

During 2007–2020, we conducted a cross-sectional prevalence study among patients with acute undifferentiated febrile illness to describe the burden and long-term epidemiology of rickettsioses in Cambodia. Serum samples were collected from 10,243 participants, along with epidemiologic data, information on clinical symptoms, demographic characteristics, and risk factors. A total of 802 (7.8%) participants met the definition for acute rickettsial infection after ruling out malaria, influenza, dengue, and chikungunya; 557 (5.4%) cases were typhus, 154 (1.5%) spotted fever, and 136 (1.3%) scrub typhus. Overall seroprevalence was 18.1% (1,857/10,243). Increased age, residence in urban settings, and recent travel to forests were significantly associated with rickettsial infection. Symptoms significantly associated with infection included rash, vomiting, and skin lesions. Our results confirm the underlying burden of rickettsioses and associated risk factors in Cambodia and highlight the need for accessible diagnostics and clinical guidance that consider rickettsioses when treating persons with acute undifferentiated febrile illness.

Rickettsioses are vectorborne bacterial infections caused by obligate, intracellular, gram-negative coccobacilli belonging to the genera *Rickettsia* and *Orientia*, which are transmitted to humans through arthropods such as ticks, fleas, mites, and lice (*1*–*4*). Transmission occurs globally and is influenced by varying factors, such as vector populations, ecology, and human activities (*5*–*7*).

The 3 main rickettsioses groups are typhus group (TG), consisting of endemic murine typhus (*Rickettsia typhi*) and the rarely occurring epidemic typhus (*R. prowazekii*); spotted fever group (SFG), consisting of multiple *Rickettsia* species (e.g., *R. rickettsii, R. conorii, R. felis)*; and the scrub typhus group (STG) (e.g., *Orientia tsutsugamushi*) (*2*,*8*). Infection is associated with a broad range of symptoms, often including an acute nonspecific febrile illness accompanied by headache, myalgia, nausea, and rash (*9*–*11*). In scrub typhus and tickborne SFG infections, an eschar at the bite site might also be observed (*9*,*10*). Although most symptomatic infections lead to mild or moderate illness, some cases can be severe and life-threatening if left untreated (*11*–*14*).

Throughout Southeast Asia, rickettsioses are a leading cause of acute febrile illness and disproportionately affect poorer communities (*1*,*10*,*15*,*16*). Despite being readily treatable with antibiotic therapy, particularly during the early course of infection, rickettsioses often remain underdiagnosed and subsequently undertreated (*1*,*10*,*13*,*17*). A lack of appropriate point-of-care diagnostics and the often nonspecific clinical manifestations associated with infection further complicate definitive diagnosis (*1*,*14*,*17*–*19*). The substantial public health concerns associated with rickettsioses (*1*,*20*–*22*) reflect the need to elucidate the true burden of disease and the associated population risk profile to support effective clinical management and public health responses.

In Cambodia, as part of a long-term health facility–based disease surveillance initiative established in 2006 by the Royal Cambodian Ministry of Health, standardized laboratory testing procedures were conducted on healthcare-seeking patients with acute undifferentiated febrile illness (AUFI) (*23*–*25*). Aims of this passive surveillance included using AUFI seroprevalence surveillance data to describe the burden and long-term epidemiology of rickettsioses among AUFI patients in Cambodia.

## Methods

### Study Site Selection, Design, and Participant Enrollment

**Figure 1:**
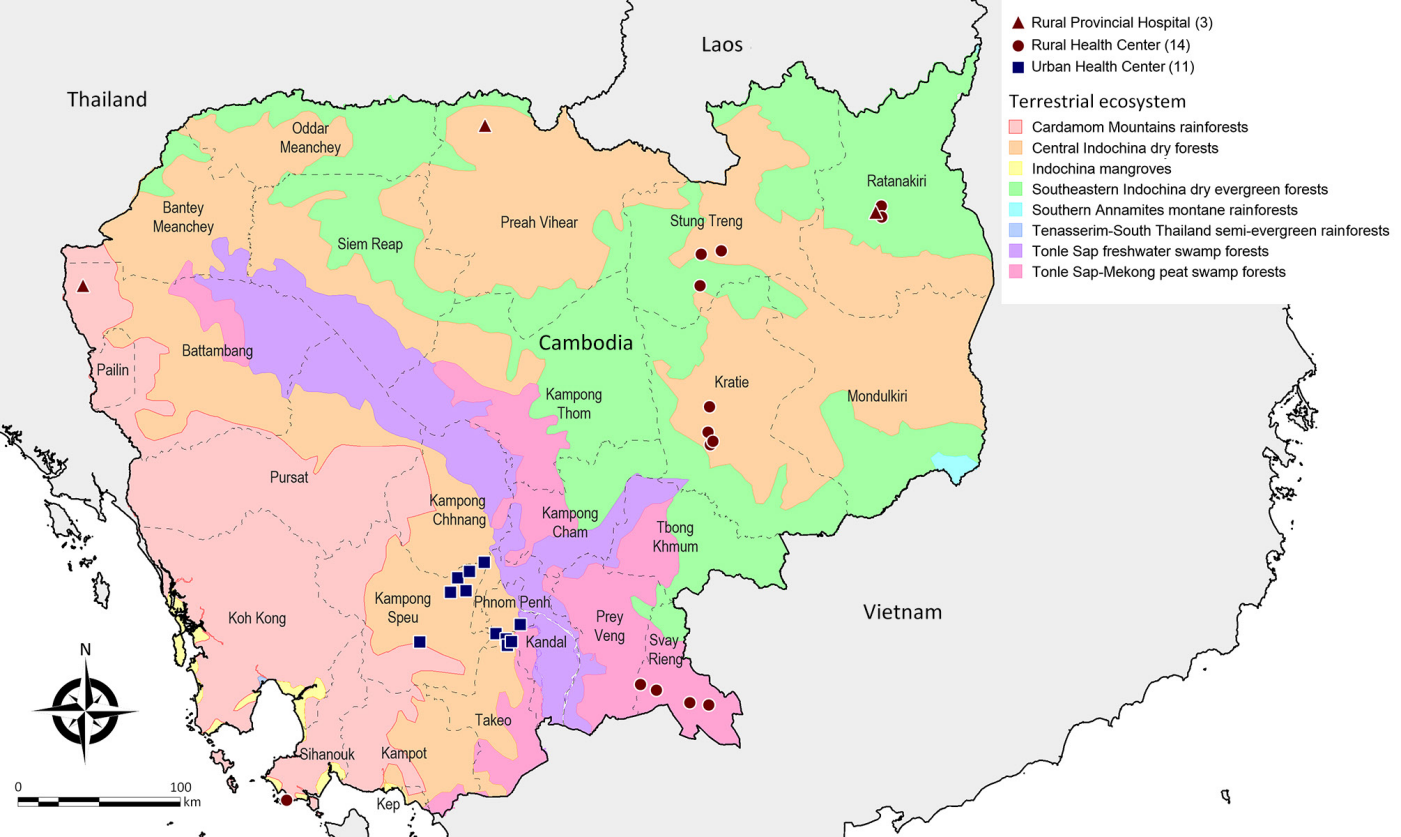
Study site locations of urban and rural health facilities included in cross-sectional prevalence study of clinical manifestations, risk factors, and disease burden of rickettsiosis, Cambodia, 2007–2020, overlayed on top of terrestrial ecoregions of Cambodia.

We conducted a cross-sectional prevalence study among participants with AUFI symptoms at selected health facilities throughout Cambodia during January 2007–December 2020. The Cambodian Ministry of Health selected 28 health facilities from 9 provinces located within 4 terrestrial ecoregions (*26*) to represent a diversity of geographic locations throughout the country. Those facilities included 3 rural provincial hospitals and 14 rural health centers, together with 11 urban health centers primarily located around the capital, Phnom Penh (Figure 1). We collected data continuously throughout the course of the study during both the dry season (November–April) and wet season (May–October). We used terrestrial ecoregions, defined as standardized large land areas classified by distinct biogeographic groups of species and ecosystems, to describe ecologic zones (*26*).

In this study, we defined AUFI as acute onset of fever lasting >24 hours but <10 days where, even after comprehensive clinical history and physical examination, a distinct etiology could not be identified as previously described (*24*,*25*,*27*). Persons >2 years of age experiencing AUFI symptoms with an oral or tympanic temperature >38°C or axillary temperature >37.5°C were eligible. As part of the AUFI surveillance initiative, patients who tested negative for influenza (by nasal swab or rapid test), malaria (by microscopy or rapid test), dengue (by rapid test or laboratory-confirmed diagnosis, including PCR or serologic assay), or chikungunya (by PCR, serologic assay, or both), as previously described (*23*–*25*,*28*), were tested for rickettsioses. Patients positive for 1 of those more common etiologies were not evaluated for rickettsioses.

We asked eligible patients to complete a questionnaire to capture demographic information such as gender, employment status, residential address, and contact details. As part of an acute-stage sampling phase, participants underwent a medical history and physical examination, and blood samples were collected for laboratory testing. An acute clinical assessment questionnaire (Appendix Figure 1) was also completed that detailed travel history or any time spent working in forested areas in the previous 2 months before assessment. Participants were asked to return 14–30 days later to complete a follow-up medical examination and provide a convalescent blood sample for laboratory analysis. If participants did not return for their scheduled convalescent visit, study-associated healthcare staff visited the participant’s place of residence to conduct follow-up assessment and specimen collection.

### Laboratory Analysis

After collection, we labeled blood samples and sent them to Phnom Penh for processing. Serum was separated from the whole blood specimen, aliquoted into prelabeled cryovials, and stored at –70°C or in liquid nitrogen. We performed rickettsioses antibody testing on serum samples using IgG in-house ELISAs developed by the Naval Medical Research Center (Silver Spring, Maryland, USA). The assays targeted antibodies against specific rickettsial antigens of the typhus group (*R. typhi*, Wilmington strain), scrub typhus group (*O. tsutsugamushi*, Gilliam, Kato, and Karp strains), and spotted fever group (*R. conorii*), as previously described (*25*,*29*–*31*). Positive and negative controls for each assay were derived from pooled serum samples collected from participants in the Cambodia AUFI surveillance initiative with net optical density (OD) values of <0.2 (negative) and >1.0 (positive). For initial screening, we tested convalescent samples at 1:100 dilution and scored them as positive if they yielded a net OD of >0.5 for any of the 3 antigen preparations. All screen-positive serum samples were titrated at 4 dilutions of 1:100, 1:400, 1:1,600, and 1:6,400. We considered samples positive when the cumulative net OD value for the 4 dilutions was >1,000. Titers for the positive samples were determined to be the inverse of the highest dilution that gave a net OD of >0.2. Samples that achieved an OD of >0.5 at screening but failed to be confirmed by titration were reported as negative for all later analyses. We then paired convalescent samples identified as IgG-positive with their corresponding acute serum samples and retested to identify a positive rickettsial infection.

### Definition of a Positive Rickettsial Infection

In this study, we defined a positive rickettsial infection as either a 4-fold increase in titer from acute to convalescent sample or a result change of negative in the acute sample to positive in the convalescent sample, considered indicative of seroconversion. We defined previous exposure (seroprevalence) as any participant with a seropositive result in either the acute or convalescent sample, regardless of seroconversion.

### Data Management and Statistical Analysis

We matched and double-entered data into Access (Microsoft, https://www.microsoft.com) and performed statistical analysis using R version 4.3.0 (The R Project for Statistical Computing, https://www.r-project.org). We used the Pearson χ^2^ test to analyze frequency of categorical variables and generalized linear models for binomial regression analysis to calculate odds ratios (ORs) and 95% CIs to measure the association between rickettsioses and recorded symptoms, demographics, and environmental characteristics. We also analyzed potential interactions between key variables including urban or rural status and terrestrial ecosystems.

### Ethical Clearance and Consent

We obtained written informed consent from eligible participants >18 years of age or the parent/legal guardian of eligible nonadult participants <18 years of age. Ethics approval was obtained by the Kingdom of Cambodia’s National Ethics Committee for Health Research (208 NECHR) and the United States Naval Medical Research Center (NAMRU2.2012.0001), in compliance with all applicable federal regulations governing the protection of human subjects.

## Results

**Figure 2:**
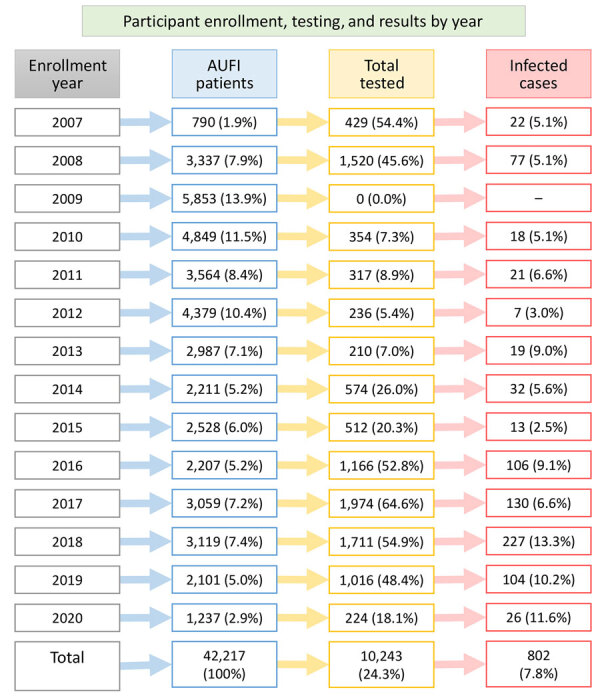
Flowchart of patients enrolled in rickettsioses cross-sectional prevalence study of clinical manifestations, risk factors, and disease burden of rickettsiosis, Cambodia, 2007–2020. Columns indicate percentage of total AUFI patents (blue), percentage of participants tested for rickettsioses (yellow), and percentage of infected persons detected per year (red). No testing was conducted in 2009. AUFI, acute undifferentiated febrile illness.

We enrolled a total of 42,221 participants during 2007–2020, of whom 29,982 (71.0%) provided paired acute-convalescent blood samples (Figure 2). In accordance with the study testing protocol, we tested 10,243 (34.2%) paired samples for rickettsioses after seeing the results of rapid and laboratory testing; we did not test 9,340 (31.1%) participants because they tested positive for influenza, malaria, dengue, or chikungunya (Figure 2). We did not test the remaining 10,399 (34.7%) participants because of supply limitations and logistical challenges experienced during the study, as well as because of host country operational priorities, including no participants being tested for rickettsioses in 2009 because of a reallocation of resources to address the swine influenza pandemic. Participants tested for rickettsioses were 2–90 years of age; the mean age was 24.2 (SD 17.3) years. The median number of tested participants per year was 512 (mean 787.9 [SD 619.6]) (Appendix Table 1).

We identified a total of 847 rickettsial infections among 802 (7.8%) participants during the study. Of those infections, most belonged to the TG (65.8%, n = 557), followed by the SFG (18.2%, n = 154), and STG (16.0%, n = 136) (Table 1). We detected co-infections with multiple rickettsioses in 44 (0.4%) of the 10,243 tested participants, including 1 participant who tested positive for all 3 rickettsial groups (Appendix Table 2). We recorded significant positive associations between TG and STG (OR 2.36 [95% CI 1.34–3.88]), SFG and TG (OR 2.50 [95% CI 1.49–3.97]), and STG and SFG (OR 6.13 [95% CI 3.06–11.1]) infections (Appendix Table 3). Among the 10,243 participants tested, 1,857 (18.1%) recorded a positive result in either the acute or convalescent sample, indicative of rickettsioses exposure, including 1,068 (10.4%) recording positive exposures to TG, 596 (5.8%) to SFG, and 451 (4.4%) to STG.

**Table 1:** Rickettsial infections detected by type and diagnostic method among patients with acute undifferentiated febrile illness tested for rickettsioses at health facilities as part of study of rickettsiosis in Cambodia, 2007–2020*

Group	Result	Diagnostic method, no. (%) patients		
		4-fold increase	Seroconversion	Seroconversion or 4-fold increase
STG	Negative	10,211 (99.7)	10,139 (99.0)	10,107 (98.7)
	Positive	32 (0.3)	104 (1.0)	136 (1.3)
TG	Negative	10,176 (99.3)	9,753 (95.2)	9,686 (94.6)
	Positive	67 (0.7)	490 (4.8)	557 (5.4)
SFG	Negative	10,221 (99.8)	10,111 (98.7)	10,089 (98.5)
	Positive	22 (0.2)	132 (1.3)	154 (1.5)

*SFG, spotted fever group; STG, scrub typhus group; TG, typhus group.

**Figure 3:**
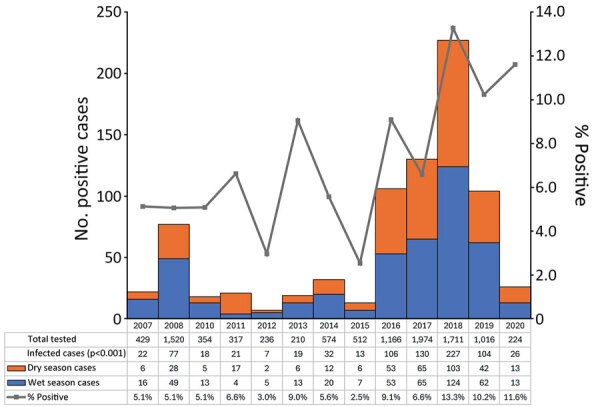
Detected rickettsial infections by year and season and yearly percentage of rickettsioses-positive patients with acute undifferentiated febrile illness in cross-sectional prevalence study of clinical manifestations, risk factors, and disease burden of rickettsiosis, Cambodia, 2007–2020. p values determined using the Pearson χ^2^ test. The year 2009 is omitted because no testing was conducted.

We identified a significant association between year and number of rickettsial infections detected over the 14-year study period (p<0.001). Most rickettsial infections were detected during 2016–2019 (70.7%, n = 567); the highest number of cases (n = 227) and proportion of positive tests by year (13.3%) occurred in 2018 (Figure 3).

Most rickettsial infections were detected among study participants >15 years of age (83.3%, n = 684), which had a recorded test positivity percentage of 11.2% (684/6,132); percentage of test positivity in participants <15 years of age (14.7%, n = 118) was 2.9% (118/4,111) (Table 2; Appendix Tables 4, 5). The highest overall percentages of positivity for rickettsial infections were recorded among participants >46 years (14.7%, 216/1,468) and 36–45 (14.2%, 156/1,096) years of age (Appendix Figure 2). In participants who reported travel to the forest, 14.8% (406/2,735) tested positive, whereas 12.0% (165/1,375) of participants who reported travel outside their home region tested positive. Similarly, high test-positivity percentages for TG infections were recorded within those categories; 11.9% (326/2,735) of participants who reported traveling to the forest tested positive for TG infections, and 9.0% (124/1,371) of participants who reported travel outside their home regions tested positive. A strong positive association was observed among participants testing positive for TG and traveling to the forest (OR 4.26 [95% CI 3.58–5.08]). 

**Table 2:** Characteristics of study participants and seroprevalence of rickettsial infection by type among patients with acute undifferentiated febrile illness tested for rickettsioses at health facilities as part of study of rickettsiosis in Cambodia, 2007–2020*

Category	Total tested, no. (%)‡	Negative, no. (%)†	Positive, no. (%)†			
			STG	TG	SFG	All rickettsiae
Participants	10,243 (100)	9,441 (92.1)	136 (1.3)	557 (5.4)	154 (1.5)	802 (7.8)
Age group, y						
<15	4,111 (40.1)	3,993 (97.1)	28 (0.7)	74 (1.8)	22 (0.5)	118 (2.9)
16–25	1,805 (17.6)	1,637 (90.7)	36 (2.0)	107 (5.9)	38 (2.1)	168 (9.3)
26–35	1,763 (17.2)	1,619 (91.8)	17 (1.0)	97 (5.5)	36 (2.0)	144 (8.2)
36–45	1,096 (10.7)	940 (85.8)	13 (1.2)	120 (10.9)	32 (2.9)	156 (14.2)
>46	1,468 (14.3)	1,252 (85.3)	42 (2.9)	159 (10.8)	26 (1.8)	216 (14.7)
Sex						
F	4,936 (48.2)	4,601 (93.2)	68 (1.4)	221 (4.5)	62 (1.3)	335 (6.8)
M	5,307 (51.8)	4,840 (91.2)	68 (1.3)	336 (6.3)	92 (1.7)	467 (8.8)
Education	6,182 (60.4)	5,653 (91.4)	91 (1.5)	376 (6.1)	86 (1.4)	529 (8.6)
Lower primary school	2,224 (21.7)	2,062 (92.7)	29 (1.3)	110 (4.9)	38 (1.7)	162 (7.3)
Primary school	938 (9.2)	875 (93.3)	6 (0.6)	40 (4.3)	19 (2.0)	63 (6.7)
Lower secondary school	816 (8.0)	770 (94.4)	10 (1.2)	29 (3.6)	11 (1.3)	46 (5.6)
High school	83 (0.8)	81 (97.6)	0 (0)	2 (2.4)	0 (0)	2 (2.4)
Diploma or university						
Employment status						
Unemployed	8,907 (87.0)	8,164 (91.7)	115 (1.3)	521 (5.8)	149 (1.7)	743 (8.3)
Employed	1,336 (13.0)	1,277 (95.6)	21 (1.6)	36 (2.7)	5 (0.4)	59 (4.4)
Season						
Dry, Nov–Apr	4,497 (43.9)	4,139 (92.0)	49 (1.1)	251 (5.6)	78 (1.7)	358 (8.0)
Wet, May–Oct	5,746 (56.1)	5,302 (92.3)	87 (1.5)	306 (5.3)	76 (1.3)	444 (7.7)
Area						
Rural	5,331 (52.0)	4,875 (91.4)	67 (1.3)	330 (6.2)	70 (1.3)	456 (8.6)
Urban	4,912 (48.0)	4,566 (93.0)	69 (1.4)	227 (4.6)	84 (1.7)	346 (7.0)
Had traveled						
No	8,872 (86.6)	8,235 (92.8)	115 (1.3)	433 (4.9)	121 (1.4)	637 (7.2%)
Yes	1,371 (13.4)	1,206 (88.0)	21 (1.5)	124 (9.0)	33 (2.4)	165 (12.0%)
Traveled to forest						
No	7,508 (73.3)	7,112 (94.7)	97 (1.3)	231 (3.1)	100 (1.3)	396 (5.3)
Yes	2,735 (26.7)	2,329 (85.2)	39 (1.4)	326 (11.9)	54 (2.0)	406 (14.8)
Terrestrial ecosystem						
Southeastern Indochina dry evergreen forests	869 (8.5)	840 (96.7)	7 (0.8)	10 (1.2)	13 (1.5)	29 (3.3)
Cardamom Mountains rain forests	292 (2.8)	287 (98.3)	2 (0.7)	3 (1.0)	1 (0.3)	5 (1.7)
Central Indochina dry forests	6,791 (66.3)	6,135 (90.3)	95 (1.4)	482 (7.1)	114 (1.7)	656 (9.7)
Tonle Sap-Mekong peat swamp forests	2,291 (22.4)	2,179 (95.1)	32 (1.4)	62 (2.7)	26 (1.1)	112 (4.9)
Antibiotic use in past 30 d						
No	9,736 (95.0)	8,979 (92.2)	129 (1.3)	523 (5.4)	149 (1.5)	757 (7.8)
Yes	507 (5.0)	462 (91.1)	7 (1.4)	34 (6.7)	5 (1.0)	45 (8.9)

*SFG, spotted fever group; STG, scrub typhus group; TG, typhus group. †Percentages of participants by category. ‡Percentages of total participants.

All persons in age groups >15 years had significantly higher odds of infection than did participants <15 years of age; persons in the 36–45-year age group had the highest likelihood (OR 4.17 [95% CI 3.14–5.54]), followed by the >46-year group (OR 4.02 [95% CI 3.07–5.26), the 16–25-year age group (OR 3.68 [95% CI 2.79–4.86), and the 26–35-year age group (OR 3.06 [95% CI 2.30–4.06]) (Table 3). In addition to age and forest exposure (OR 1.87 [95% CI 1.52–2.30]); other risk factors included participants testing positive in health facilities located in Central Indochina dry forest (OR 3.68 [95% CI 2.51–5.60]) and Tonle Sap-Mekong peat swamp forest ecoregions (OR 1.74 [95% CI 1.03–2.98]), and urban areas (OR 1.95 [95% CI 1.29–2.99). In addition to education, significant negative associations were recorded among employed participants (OR 0.69 [95% CI 0.50–0.94). We did not observe any significant association between rickettsial infection and gender, general travel, or season (wet or dry) in this study (Appendix Table 6).

**Table 3:** Association between key participant characteristics and rickettsial infection among patients with acute undifferentiated febrile illness who tested positive for rickettsioses as part of study of rickettsiosis in Cambodia, 2007–2020*

Characteristic	OR (95% CI)	p value
Year	1.04 (1.02–1.07)	0.001
Age group, y		
<15	Referent	
16–25	3.68 (2.79–4.86)	<0.001
26–35	3.06 (2.30–4.06)	<0.001
36–45	4.17 (3.14–5.54)	<0.001
>46	4.02 (3.07–5.26)	<0.001
Sex		
F	Referent	
M	1.16 (0.99–1.36)	0.072
Education		
Lower primary school	Referent	
Primary school	0.77 (0.63–0.95)	0.014
Lower secondary school	0.70 (0.52–0.94)	0.019
High school	0.56 (0.39–0.77)	<0.001
Diploma or university	0.23 (0.04–0.77)	0.047
Employment status		
Unemployed	Referent	
Employed	0.69 (0.50–0.94)	0.022
Season		
Dry, Nov–Apr	Referent	
Wet, May–Oct	0.97 (0.84–1.13)	0.7
Area		
Rural	Referent	
Urban	1.95 (1.29–2.99)	0.002
Terrestrial ecosystem		
Southeastern Indochina dry evergreen forests	Referent	
Cardamom Mountains rain forests	0.90 (0.30–2.21)	0.8
Central Indochina dry forests	3.68 (2.51–5.60)	<0.001
Tonle Sap-Mekong peat swamp forests	1.74 (1.03–2.98)	0.041
Influence of rural/urban setting within terrestrial ecosystem†		
Urban/Cardamom Mountains rain forests		
Urban/Central Indochina dry forests	0.52 (0.32–0.85)	0.009
Urban/Tonle Sap-Mekong peat swamp forests		
Had traveled		
No	Referent	
Yes	0.79 (0.62–1.01)	0.061
Traveled to forest		
No	Referent	
Yes	1.87 (1.52–2.30)	<0.001
Antibiotic use in past 30 d		
No	Referent	
Yes	1.02 (0.72–1.41)	>0.9

*Determined by generalized linear model for binomial regression. OR, odds ratio. †Reference group is rural location (data not listed) for respective urban ecosystems.

Positive symptom associations included rash (adjusted OR [aOR] 2.21 [95% CI 1.63–2.97]), vomiting (aOR 1.64 [95% CI 1.29–2.07]), and skin lesions (aOR 1.63 [95% CI 1.25–2.12]) (Table 4). Among participants tested for rickettsioses, 23.1% (129/559) of persons with skin lesions, and 22.8% (76/334) of persons with rash tested positive for a rickettsial infection (Table 4).

**Table 4:** Association between reported symptoms and rickettsial infection among patients with acute undifferentiated febrile illness who tested positive for rickettsioses as part of study of rickettsiosis in Cambodia, 2007–2020*

Symptom	Present	Rickettsial infection								
		Total, n = 10,243	Negative, n = 9,441	Positive, n = 802		Unadjusted†			Adjusted‡	
						OR (95% CI)	p value		OR (95% CI)	p value
Fever	Yes	10,243	9,441 (92.2)	802 (7.8)						
Malaise	No	3,373	3,204 (95.0)	169 (5.0)		Referent			Referent	
	Yes	6,870	6,237 (90.8)	633 (9.2)		1.92 (1.62–2.30)	<0.001		0.76 (0.60–0.96)	0.019
Chills	No	4,475	4,260 (95.2)	215 (4.8)		Referent			Referent	
	Yes	5,768	5,181 (89.8)	587 (10.2)		2.24 (1.91–2.64)	<0.001		1.39 (1.16–1.68)	<0.001
Joint Pain	No	7,359	6,921 (94.1)	438 (5.9)		Referent			Referent	
	Yes	2,884	2,520 (87.4)	364 (12.6)		2.28 (1.97–2.64)	<0.001		1.15 (0.95–1.38)	0.2
Nausea	No	7,594	7,104 (93.6)	490 (6.4)		Referent			Referent	
	Yes	2,649	2,337 (88.2)	312 (11.8)		1.94 (1.67–2.25)	<0.001		0.79 (0.62–1.00)	0.056
Vomit	No	8,347	7,820 (93.7)	527 (6.3)		Referent			Referent	
	Yes	1,896	1,621 (85.5)	275 (14.5)		2.52 (2.15–2.94)	<0.001		1.64 (1.29–2.07)	<0.001
Abdominal cramps	No	9,083	8,349 (91.9)	734 (8.1)		Referent			Referent	
	Yes	1,160	1,092 (94.1)	68 (5.9)		0.71 (0.54–0.91)	0.008		0.77 (0.55–1.04)	0.10
Diarrhea	No	9,813	9,048 (92.2)	765 (7.8)		Referent			Referent	
	Yes	430	393 (91.4)	37 (8.6)		1.11 (0.78–1.55)	0.5		1.57 (1.02–2.38)	0.036
Bloody stool	No	10,203	9,403 (92.2)	800 (7.8)		Referent			Referent	
	Yes	40	38 (95.0)	2 (5.0)		0.62 (0.10–2.02)	0.5		0.52 (0.08–1.87)	0.4
Cough	No	3,362	3,099 (92.2)	263 (7.8)		Referent			Referent	
	Yes	6,881	6,342 (92.2)	539 (7.8)		0.86–1.17	>0.9		0.95 (0.77–1.18)	0.7
Headache	No	1,615	1,566 (97.0)	49 (3.0)		Referent			Referent	
	Yes	8,628	7,875 (91.3)	753 (8.7)		3.06 (2.30–4.15)	<0.001		1.47 (1.07–2.07)	0.020
Sore throat	No	4,348	4,040 (92.9)	308 (7.1)		Referent			Referent	
	Yes	5,895	5,401 (91.6)	494 (8.4)		1.20 (1.04–1.39)	0.016		0.99 (0.81–1.21)	>0.9
Muscle aches	No	6,223	5,944 (95.5)	279 (4.5)		Referent			Referent	
	Yes	4,020	3,497 (87.0)	523 (13.0)		3.19 (2.74–3.71)	<0.001		1.39 (1.12–1.74)	0.004
Shortness of breath	No	9,430	8,681 (92.1)	749 (7.9)		Referent			Referent	
	Yes	813	760 (93.5)	53 (6.5)		0.81 (0.60–1.07)	0.15		0.84 (0.62–1.13)	0.3
Rash	No	9,909	9,183 (92.7)	726 (7.3)		Referent			Referent	
	Yes	334	258 (77.3)	76 (22.7)		3.73 (2.84–4.84)	<0.001		2.21 (1.63–2.97)	<0.001
Lesion	No	9,684	9,011 (93.1)	673 (6.9)		Referent			Referent	
	Yes	559	430 (76.9)	129 (23.1)		4.02 (3.24–4.95)	<0.001		1.63 (1.25–2.12)	<0.001
Seizure	No	10,172	9,375 (92.2)	797 (7.8)		Referent			Referent	
	Yes	71	66 (93.0)	5 (7.0)		0.89 (0.31–2.01)	0.8		1.00 (0.34–2.35)	>0.9
Other symptoms	No	9,670	8,881 (91.8)	789 (8.2)		Referent			Referent	
	Yes	573	560 (97.7)	13 (2.3)		0.26 (0.14–0.44)	<0.001		0.36 (0.19–0.60)	<0.001

*Values are no. (%) except as indicated. OR, odds ratio. †Unadjusted generalized linear model for binomial regression. ‡Generalized linear model for binomial regression adjusted by age, gender, and enrollment year.

## Discussion

Using laboratory-level diagnostics and associated patient and community level variables, this study provides a unique opportunity to describe the variable burden and characteristics of rickettsial infection in Cambodia over a 14-year period. The overall rickettsioses test-positivity percentage of 7.8% observed in this study demonstrates a notable burden of disease among febrile patients seeking treatment who had tested negative for influenza, malaria, dengue, and chikungunya. Similarly, the positive detection of antibodies in 18.1% of all tested participants suggests a significant level of exposure to rickettsioses among the population of Cambodia. Wide variances in rickettsioses seroprevalence proportions are noted in the literature (5%–30%); differences are often explained by geography, study design, targeted population immunological profiles, diagnostic approaches used, and thresholds or clinical definitions applied, likely contributing to this wide range (*10*,*32*). However, this study recorded a higher prevalence of TG cases (5.4%) than STG cases (1.3%), in contrast to previous studies from adjacent countries such as Thailand (9.0% TG vs. 9.8% STG), Vietnam (3.7% TG vs. 26.4% STG), and Laos (9.6% TG vs. 14.8% STG) (*33*–*35*). Of note, the prevalence of confirmed STG cases (1.3%) and overall STG seroprevalence (4.4%) in this study were lower than that observed in studies referenced from neighboring countries but are similar to those reported for similar AUFI studies in Cambodia (*36*). The discordance might relate to vector dynamics, disease reservoirs, or differences among target population risk profiles across studies, which will require further research to confirm the causative factors driving these variances.

Although we did not compare the prevalence of rickettsioses to influenza, malaria, dengue, and chikungunya, our results indicated that almost 1 in 10 cases of AUFI in this study were likely caused by rickettsiosis (after those more common etiologies had been ruled out). Given rickettsial infections are known to be chronically underdiagnosed, those findings highlight a clear need for accessible point-of-care diagnostics and effective surveillance. Similarly, strengthening awareness and education among healthcare providers and ensuring appropriate antibiotic treatments are available is critical to addressing rickettsiosis in Cambodia.

The number of positive cases detected over time was shown to differ in this study; yearly test positivity percentages ranged from 2.5% in 2015 to 11.6% in 2020, illustrating variable transmission dynamics. Such longitudinal variability indicates a need to incorporate effective surveillance into routine health system practice to adequately monitor transmission and identify potential disease outbreaks. Challenges associated with early diagnosis, variable transmission rates, and the potential for serious and life-threatening illness, when paired with exposure and infection rates observed in this study, highlight the public health impact of rickettsioses in Cambodia. The final 3 years of the study recorded the 3 highest test-positivity percentages recorded over the 14-year study period, which also saw positivity percentages for rickettsial DNA as high as 36% in ectoparasites collected in southern Cambodia (*37*), indicating a potential resurgence of rickettsial infections in recent years. That period was immediately before lockdowns associated with the COVID-19 pandemic and might not be reflective of current patterns, warranting the investigation of contemporary epidemiologic data.

Consistent with previous studies from neighboring countries, including Thailand, Laos, and Malaysia (*33*,*38*,*39*), our findings demonstrated a significant association between increasing age and rickettsial infection. Higher case rates among adults than in children suggests potential increased exposure among participants >15 years of age to environmental settings favorable to the vectors and vector hosts known to transmit rickettsial bacteria, likely because of occupational or lifestyle activities. This study also identified a negative association between rickettsial infection and participants with formal employment and higher levels of education, consistent with findings from previous studies within the region examining those sociodemographic variables (*33*,*38*). Of note, we observed similar proportions of STG seroprevalence and infection between urban and rural participants in this study. Given the high percentage of the labor force engaged in the agricultural sector (32%), particularly rice production, together with Cambodia’s relatively small size and good transport network (*40*), those findings warrant further detailed investigation into the effects of occupational exposure and risk stratification on STG infection.

In addition to age across all 3 main rickettsioses groups, we identified significant associations between SFG infection and urban settings, as well as associations between infection and travel to forests and associations between the Central Indochina dry forests and Tonle Sap-Mekong peat swamp forest ecoregions with TG infection. Those findings suggest lifestyle and occupational and environmental exposure, such as living in high-density residencies or engaging in forest-based and forest-fringe–based informal occupations (such as agriculture, logging, and animal trapping), are all associated with an increased risk for specific types of infection. Providers should have a higher index of suspicion for rickettsial infection if those risk factors are present. We also found no significant difference in association between wet and dry seasons in this study, indicating that rickettsial infections should be considered as a potential etiology regardless of season.

In this study, we sought to elucidate the burden of rickettsial disease in Cambodia after ruling out more common pathogens. Consistent with other studies, significant symptoms associated with rickettsial infections observed after multivariable analysis consisted of rash, vomiting, skin lesions, headache, diarrhea, chills, and muscle aches (*10*,*19*,*41*,*42*). Despite those associations, the nonspecific nature of those symptoms is common to many febrile illnesses, emphasizing the challenges associated with the clinical diagnosis of rickettsioses, particularly in the absence of specific laboratory diagnostics (*3*,*10*,*19*). Of note, we observed a high proportion of specific gastrointestinal symptoms among infected participants. Previous research highlights a higher propensity for the misdiagnosis of rickettsial infections if gastrointestinal symptoms are prominent or no skin rash is present (*10*,*19*). Therefore, providers should not exclude rickettsioses from the differential diagnosis in the setting of vomiting or diarrhea and could consider empiric therapy if proper diagnostics are not available. The overlap in the identified symptom associations of rickettsioses with other well-known febrile illnesses in the region, such as dengue and malaria, together with relatively high proportions of rickettsial infection identified among participants tested in this study, highlights the overlapping nonspecific clinical manifestations likely seen by frontline healthcare providers. The substantial risk of underdiagnosing rickettsioses supports the development of a point-of-care diagnostic that can be used in low-resource settings.

The first limitation of this study is its reliance on deidentified passive surveillance data collected from symptomatic patients seeking care for AUFI; persons with asymptomatic and mildly symptomatic rickettsial infections might have been missed, because those patients would not seek care. Similarly, only AUFI patients who tested negative for influenza, malaria, dengue, and chikungunya were tested for rickettsioses, meaning rickettsial-infected patients co-infected with any of those diseases could have been missed in this study because of selection bias. Testing proportions might also have been influenced by seasonal factors; 19.7% of AUFI patients were tested for rickettsioses during the wet season compared with 32.1% in the dry season, possibly because of higher positivity proportions for other diseases during the wet season or seasonal dynamics of the vectors. Those factors might have led to underestimating the true prevalence of rickettsioses. Also, because most study site facilities were located within the Central Indochina dry forests and Tonle Sap-Mekong peat swamp forests ecoregions, the potential for bias associated with the uneven distribution of sites should be considered when interpreting the terrestrial ecoregion analyses. The collection of additional patient level data (such as occupation and specific participant interactions with domestic or agricultural animals), as well as household level information (such as number of persons residing in the household) and the expansion of data collection to all ecoregion types in Cambodia, should be incorporated into future surveillance efforts to fill additional knowledge gaps identified by this effort.

This long-term surveillance study has documented the rate of rickettsial infections among AUFI patients in Cambodia as ≈8% once more common etiologies were ruled out; seroprevalence was 18%. Clinical and epidemiologic risk factors identified over the 14-year study period associated with rickettsial infection, in addition to fever, include rash, skin lesions, and vomiting, as well as age, residence in urban settings, and recent travel to forests. Given the complex diagnostic challenges, clinical prognosis, and disease burden identified in this study, point-of-care diagnostics for resource-limited settings are needed. In the meantime, providers with clinical suspicion could consider empiric therapy. Additional surveillance and research are needed to better define transmission dynamics, the associated risk profiles of populations most at risk, the specific rickettsial pathogens responsible for disease (i.e., species specific molecular and serologic assays), and the influence of environmental and economic changes to inform appropriate public health interventions. Our results add to our overall understanding and awareness of rickettsioses in Cambodia and make a strong case for deploying diagnostic tools for detecting TG, SFG, and STG rickettsiae in clinical settings.

Appendix Additional information about clinical manifestations, risk factors, and disease burden of rickettsiosis, Cambodia, 2007–2020
