# Supplementary material for: Clinical Manifestations, Risk Factors, and Disease Burden of Rickettsiosis, Cambodia, 2007–2020
Source: Emerg Infect Dis. 2025 Jun;31(6):1069–80. doi: 10.3201/eid3106.241752 (PMC12123938; doi:10.3201/eid3106.241752)
Supplement: Appendix — Additional information about clinical manifestations, risk factors, and disease burden of rickettsiosis, Cambodia, 2007–2020 [file 24-1752-Techapp-s1.pdf]

Article DOI: https://doi.org/10.3201/eid3106.241752

EID cannot ensure accessibility for supplementary materials supplied by authors. Readers who have difficulty accessing supplementary content should contact the authors for assistance.

Clinical Manifestations, Risk Factors, and Disease Burden of Rickettsiosis, Cambodia, 2007–2020

Appendix

Appendix Table 1. Summary of AUFI patients and study participants tested for rickettsioses by enrolment year and season (wet and dry) as part of a cross-sectional prevalence study in Cambodia from 2007 – 2020*

	TOTAL AUFI PATIENTS			PARTICIPANTS TESTED FOR RICKETTSIA		
**Year**	**Overall**	**Dry[1]**	**Wet[1]**	**Overall[1]**	**Dry[2]**	**Wet[3]**
**2007**	790 (100.0%)	282 (35.7%)	508 (64.3%)	429 (54.4%)	225 (79.8%)	204 (40.2%)
**2008**	3,337 (100.0%)	1,053 (31.6%)	2,284 (68.4%)	1,520 (45.6%)	356 (33.8%)	1,164 (51.0%)
**2009**	5,853 (100.0%)	1,795 (30.7%)	4,058 (69.3%)	0 (0.0%)	0 (0.0%)	0 (0.0%)
**2010**	4,849 (100.0%)	1,679 (34.6%)	3,170 (65.4%)	354 (7.3%)	159 (9.5%)	195 (6.2%)
**2011**	3,564 (100.0%)	1,142 (32.0%)	2,422 (68.0%)	317 (8.9%)	278 (24.3%)	39 (1.6%)
**2012**	4,379 (100.0%)	1,596 (36.4%)	2,783 (63.6%)	236 (5.4%)	126 (7.9%)	110 (4.0%)
**2013**	2,987 (100.0%)	1,071 (35.9%)	1,916 (64.1%)	210 (7.0%)	88 (8.2%)	122 (6.4%)
**2014**	2,211 (100.0%)	1,057 (47.8%)	1,154 (52.2%)	574 (26.0%)	350 (33.1%)	224 (19.4%)
**2015**	2,528 (100.0%)	1,044 (41.3%)	1,484 (58.7%)	512 (20.3%)	327 (31.3%)	185 (12.5%)
**2016**	2,207 (100.0%)	941 (42.6%)	1,266 (57.4%)	1,166 (52.8%)	666 (70.8%)	500 (39.5%)
**2017**	3,059 (100.0%)	1,227 (40.1%)	1,832 (59.9%)	1,974 (64.6%)	969 (79.0%)	1,005 (54.9%)
**2018**	3,119 (100.0%)	1,075 (34.5%)	2,044 (65.5%)	1,711 (54.9%)	829 (77.1%)	882 (43.2%)
**2019**	2,101 (100.0%)	803 (38.2%)	1,298 (61.8%)	1,016 (48.4%)	438 (54.5%)	578 (44.5%)
**2020**	1,237 (100.0%)	677 (54.7%)	560 (45.3%)	224 (18.1%)	152 (22.5%)	72 (12.9%)
**TOTAL**	**42,221 (100%)**	**15,442 (36.6%)**	**26,779 (63.4%)**	**10,243 (24.3%)**	**4,963 (32.1%)**	**5,280 (19.7%)**

*AUFI, acute undifferentiated febrile illness.

Appendix Table 2. Summary of participant Rickettsioses co-infection status among patients with acute undifferentiated febrile illness presenting to study site health facilities and testing positive for rickettsioses as part of a cross-sectional prevalence study in Cambodia from 2007 - 2020

Infection type	Status	**Participants (n = 42,221)**
**Rickettsial Infection**	No	41,419 (98.1%)
	Yes	802 (1.9%)
**Rickettsial Co-infection**	No	42,177 (99.9%)
	Yes	44 (0.1%)
**Rickettsial Co-infection (number of groups)**	1 Group	758 (1.8%)
	2 Groups	43 (0.1%)
	3 Groups	1 (0.0%)

Appendix Table 3. Association between Rickettsial infection by group among patients with acute undifferentiated febrile illness presenting to study site health facilities and testing positive for rickettsioses as part of a cross-sectional prevalence study in Cambodia from 2007 – 2020*

		STG			TG			SFG		
Type	Status	OR	95% CI	p-value	OR	95% CI	p-value	OR	95% CI	p-value
TG	No	—	—					—	—	
	Yes	2.36	1.34, 3.88	0.001				2.50	1.49, 3.97	<0.001
SFG	No	—	—		—	—				
	Yes	6.13	3.06, 11.1	<0.001	2.50	1.49, 3.97	<0.001			
STG	No				—	—		—	—	
	Yes				2.36	1.34, 3.88	0.001	6.13	3.06, 11.1	<0.001

*Determined by generalized linear model for binomial regression. – represents the reference group of each section. OR, odds ratio; SFG, spotted fever group; STG, scrub typhus group; TG, typhus group.

Appendix Table 4. Seroprevalence of Rickettsial infection by type and participant characteristic among patients with acute undifferentiated febrile illness presenting to study site health facilities and testing positive for rickettsioses as part of a cross-sectional prevalence study in Cambodia from 2007 - 2020

Category	Characteristic	STG Sero	STG 4fold	STG All	TG Sero	TG 4fold	TG All	SFG Sero	SFG 4fold	SFG All	Rickettsia All
Age Breakdown	≤15 y (n = 4111)	30 (1.7%)	6 (0.3%)	36 (2.0%)	94 (5.2%)	13 (0.7%)	107 (5.9%)	36 (2.0%)	2 (0.1%)	38 (2.1%)	118 (2.9%)
	16–25 y (n = 1805)	13 (0.7%)	4 (0.2%)	17 (1.0%)	85 (4.8%)	12 (0.7%)	97 (5.5%)	32 (1.8%)	4 (0.2%)	36 (2.0%)	168 (9.3%)
	26–35 y (n = 1763)	10 (0.9%)	3 (0.3%)	13 (1.2%)	108 (9.9%)	12 (1.1%)	120 (11%)	28 (2.6%)	4 (0.4%)	32 (2.9%)	144 (8.2%)
	36–45 y (n = 1096)	30 (2.0%)	12 (0.8%)	42 (2.9%)	138 (9.4%)	21 (1.4%)	159 (11%)	16 (1.1%)	10 (0.7%)	26 (1.8%)	156 (14.2%)
	≥46 y (n = 1468)	53 (1.1%)	15 (0.3%)	68 (1.4%)	195 (4.0%)	26 (0.5%)	221 (4.5%)	54 (1.1%)	8 (0.2%)	62 (1.3%)	216 (14.7%)
Gender	Female (n = 4936)	51 (1.0%)	17 (0.3%)	68 (1.3%)	295 (5.6%)	41 (0.8%)	336 (6.3%)	78 (1.5%)	14 (0.3%)	92 (1.7%)	335 (6.8%)
	Male (n = 5307)	70 (1.1%)	21 (0.3%)	91 (1.5%)	342 (5.5%)	34 (0.5%)	376 (6.1%)	71 (1.1%)	15 (0.2%)	86 (1.4%)	467 (8.8%)
Education	Lower primary school (n = 6182)	20 (0.9%)	9 (0.4%)	29 (1.3%)	92 (4.1%)	18 (0.8%)	110 (4.9%)	35 (1.6%)	3 (0.1%)	38 (1.7%)	529 (8.6%)
	Primary school (n = 2224)	6 (0.6%)	0 (0%)	6 (0.6%)	33 (3.5%)	7 (0.7%)	40 (4.3%)	17 (1.8%)	2 (0.2%)	19 (2.0%)	162 (7.3%)
	Lower secondary school (n = 938)	8 (1.0%)	2 (0.2%)	10 (1.2%)	22 (2.7%)	7 (0.9%)	29 (3.6%)	9 (1.1%)	2 (0.2%)	11 (1.3%)	63 (6.7%)
	High school (n = 816)	0 (0%)	0 (0%)	0 (0%)	1 (1.2%)	1 (1.2%)	2 (2.4%)	0 (0%)	0 (0%)	0 (0%)	46 (5.6%)
	Diploma or university (n = 83)	87 (1.0%)	28 (0.3%)	115 (1.3%)	461 (5.2%)	60 (0.7%)	521 (5.8%)	131 (1.5%)	18 (0.2%)	149 (1.7%)	2 (2.4%)
Employment status	Unemployed (n = 8907)	17 (1.3%)	4 (0.3%)	21 (1.6%)	29 (2.2%)	7 (0.5%)	36 (2.7%)	1 (<0.1%)	4 (0.3%)	5 (0.4%)	743 (8.3%)
	Employed (n = 1336)	43 (0.8%)	10 (0.2%)	53 (1.0%)	140 (2.5%)	21 (0.4%)	161 (2.9%)	43 (0.8%)	5 (<0.1%)	48 (0.9%)	59 (4.4%)
Marriage status	Single (n = 5492)	53 (1.2%)	17 (0.4%)	70 (1.6%)	328 (7.3%)	41 (0.9%)	369 (8.2%)	87 (1.9%)	17 (0.4%)	104 (2.3%)	248 (4.5%)
	Married (n = 4483)	8 (3.3%)	5 (2.1%)	13 (5.4%)	21 (8.8%)	5 (2.1%)	26 (11%)	2 (0.8%)	0 (0%)	2 (0.8%)	513 (11.4%)
	Widowed (n = 240)	0 (0%)	0 (0%)	0 (0%)	1 (3.6%)	0 (0%)	1 (3.6%)	0 (0%)	0 (0%)	0 (0%)	40 (16.7%)
	Divorced (n = 28)	37 (0.8%)	12 (0.3%)	49 (1.1%)	220 (4.9%)	31 (0.7%)	251 (5.6%)	68 (1.5%)	10 (0.2%)	78 (1.7%)	1 (3.6%)
Season	Dry (Nov–Apr) (n = 4497)	67 (1.2%)	20 (0.3%)	87 (1.5%)	270 (4.7%)	36 (0.6%)	306 (5.3%)	64 (1.1%)	12 (0.2%)	76 (1.3%)	358 (8.0%)
	Wet (May–Oct) (n = 5746)	60 (1.1%)	7 (0.1%)	67 (1.3%)	308 (5.8%)	22 (0.4%)	330 (6.2%)	57 (1.1%)	13 (0.2%)	70 (1.3%)	444 (7.7%)
Area	Rural (n = 5331)	44 (0.9%)	25 (0.5%)	69 (1.4%)	182 (3.7%)	45 (0.9%)	227 (4.6%)	75 (1.5%)	9 (0.2%)	84 (1.7%)	456 (8.6%)
	Urban (n = 4912)	88 (1.0%)	27 (0.3%)	115 (1.3%)	380 (4.3%)	53 (0.6%)	433 (4.9%)	100 (1.1%)	21 (0.2%)	121 (1.4%)	346 (7.0%)
Had traveled	No (n = 8872)	16 (1.2%)	5 (0.4%)	21 (1.5%)	110 (8.0%)	14 (1.0%)	124 (9.0%)	32 (2.3%)	1 (<0.1%)	33 (2.4%)	637 (7.2%)
	Yes (n = 1371)	72 (1.0%)	25 (0.3%)	97 (1.3%)	191 (2.5%)	40 (0.5%)	231 (3.1%)	82 (1.1%)	18 (0.2%)	100 (1.3%)	165 (12.0%)
Traveled to forest	No (n = 7508)	32 (1.2%)	7 (0.3%)	39 (1.4%)	299 (11%)	27 (1.0%)	326 (12%)	50 (1.8%)	4 (0.1%)	54 (2.0%)	396 (5.3%)
	Yes (n = 2735)	4 (0.5%)	3 (0.3%)	7 (0.8%)	9 (1.0%)	1 (0.1%)	10 (1.2%)	9 (1.0%)	4 (0.5%)	13 (1.5%)	406 (14.8%)
Terrestrial Ecosystem	Southeastern Indochina dry evergreen forests (n = 869)	0 (0%)	2 (0.7%)	2 (0.7%)	2 (0.7%)	1 (0.3%)	3 (1.0%)	0 (0%)	1 (0.3%)	1 (0.3%)	29 (3.3%)

Category	Characteristic	STG Sero	STG 4fold	STG All	TG Sero	TG 4fold	TG All	SFG Sero	SFG 4fold	SFG All	Rickettsia All
	Cardamom Mountains rain forests (n = 292)	71 (1.0%)	24 (0.4%)	95 (1.4%)	422 (6.2%)	60 (0.9%)	482 (7.1%)	97 (1.4%)	17 (0.3%)	114 (1.7%)	5 (1.7%)
	Central Indochina dry forests (n = 6791)	29 (1.3%)	3 (0.1%)	32 (1.4%)	57 (2.5%)	5 (0.2%)	62 (2.7%)	26 (1.1%)	0 (0%)	26 (1.1%)	656 (9.7%)
	Tonle Sap-Mekong peat swamp forests (n = 2291)	100 (1.0%)	29 (0.3%)	129 (1.3%)	459 (4.7%)	64 (0.7%)	523 (5.4%)	128 (1.3%)	21 (0.2%)	149 (1.5%)	112 (4.9%)
Antibiotic use in last 30 d	No (n = 9736)	4 (0.8%)	3 (0.6%)	7 (1.4%)	31 (6.1%)	3 (0.6%)	34 (6.7%)	4 (0.8%)	1 (0.2%)	5 (1.0%)	757 (7.8%)
	Yes (n = 507)	21 (0.5%)	7 (0.2%)	28 (0.7%)	65 (1.6%)	9 (0.2%)	74 (1.8%)	20 (0.5%)	2 (<0.1%)	22 (0.5%)	45 (8.9%)

*SFG, spotted fever group; STG, scrub typhus group; TG, typhus group.

Appendix Table 5. Participant age-related distribution and association with Rickettsial infection among patients with acute undifferentiated febrile illness presenting to study site health facilities and tested for rickettsioses as part of a cross-sectional prevalence study in Cambodia from 2007 – 2020*

Age (yrs)	Total Tests	Negative Result	Positive Case	Unadjusted OR	p-value
≤5	1,362	1,334	28	ref	p < 0.001
6 – 10	1,701	1,657	44	1.27	
11 – 15	1,048	1,002	46	2.19	
16 – 20	804	728	76	4.97	
21 – 25	1,001	909	92	4.82	
26 – 30	1,048	969	79	3.88	
31 – 35	715	650	65	4.76	
36 – 40	673	574	99	8.22	
41 – 45	423	366	57	7.42	
46 – 50	528	450	78	8.26	
51 – 55	341	299	42	6.69	
56 – 60	259	220	39	8.45	
61 - 65	167	133	34	12.18	
≥66	173	150	23	7.31	
Total	10,243	9,441	802		

*OR, odds ratio.

Appendix Table 6. Association between key participant characteristics and Rickettsial infection by type among patients with acute undifferentiated febrile illness presenting to study site health facilities and testing positive for rickettsioses as part of a cross-sectional prevalence study in Cambodia from 2007 – 2020*

	STG			TG			SFG		
Category	OR	95% CI	p-value	OR	95% CI	p-value	OR	95% CI	p-value
Year	0.95	0.90, 1.00	0.045	1.09	1.05, 1.12	<0.001	1.00	0.95, 1.05	>0.9
Age breakdown									
<15 y	—	—		—	—		—	—	
16–25 y	4.31	2.43, 7.70	<0.001	3.13	2.21, 4.43	<0.001	4.96	2.79, 9.00	<0.001
26–35 y	2.00	1.00, 3.86	0.043	2.66	1.87, 3.78	<0.001	5.47	3.07, 9.91	<0.001
36–45 y	2.26	1.08, 4.53	0.025	3.87	2.75, 5.48	<0.001	6.83	3.76, 12.6	<0.001
≥46 yrs	4.97	2.89, 8.62	<0.001	3.55	2.55, 4.96	<0.001	4.05	2.18, 7.57	<0.001

Category	STG			TG			SFG		
	OR	95% CI	p-value	OR	95% CI	p-value	OR	95% CI	p-value
Gender									
Female	—	—		—	—		—	—	
Male	1.00	0.70, 1.43	>0.9	1.19	0.98, 1.45	0.075	1.17	0.83, 1.66	0.4
Education									
Lower primary school	—	—		—	—		—	—	
Primary school	0.71	0.44, 1.12	0.2	0.78	0.61, 0.99	0.043	1.04	0.68, 1.56	0.9
Lower secondary school	0.28	0.11, 0.62	0.004	0.70	0.48, 1.00	0.059	1.16	0.66, 1.95	0.6
High school	0.44	0.20, 0.88	0.028	0.59	0.38, 0.88	0.013	0.77	0.37, 1.45	0.4
Diploma or university	0.00	0.00, 0.00	>0.9	0.42	0.07, 1.41	0.2	0.00	0.00, 0.00	>0.9
Employment status									
Unemployed	—	—		—	—		—	—	
Employed	1.46	0.82, 2.50	0.2	0.71	0.47, 1.04	0.086	0.21	0.07, 0.48	0.001
Season									
Dry (Nov–Apr)	—	—		—	—		—	—	
Wet (May–Oct)	1.41	1.0, 2.03	0.057	0.95	0.80, 1.14	0.6	0.76	0.55, 1.04	0.089
Area									
Rural	—	—		—	—		—	—	
Urban	1.88	0.88, 4.17	0.11	1.54	0.90, 2.69	0.12	6.67	2.24, 28.7	0.003
Had traveled									
No	—	—		—	—		—	—	
Yes	1.22	0.69, 2.07	0.5	0.65	0.49, 0.87	0.004	1.29	0.77, 2.10	0.3
Traveled to forest									
No	—	—		—	—		—	—	
Yes	0.79	0.48, 1.30	0.3	2.64	2.07, 3.40	<0.001	0.66	0.42, 1.03	0.066
Terrestrial Ecosystem									
Southeastern Indochina dry evergreen forests	—	—		—	—		—	—	
Cardamom Mountains rain forests	0.97	0.14, 4.33	>0.9	1.87	0.41, 6.33	0.4	0.23	0.01, 1.23	0.2
Central Indochina dry forests	1.39	0.65, 3.45	0.4	8.52	4.69, 17.4	<0.001	0.83	0.45, 1.65	0.6
Tonle Sap-Mekong peat swamp forests	0.88	0.33, 2.57	0.8	4.49	2.10, 10.3	<0.001	0.17	0.04, 0.56	0.008
Area*Terrestrial Ecosystem									
Urban * Cardamom Mountains rain forests	N/A			N/A			N/A		
Urban * Central Indochina dry forests	0.45	0.18, 1.15	0.10	0.77	0.41, 1.41	0.4	0.16	0.04, 0.55	0.008
Urban * Tonle Sap-Mekong peat swamp forests	N/A			N/A			N/A		
Antibiotic use in last 30 d									
No	—	—		—	—		—	—	
Yes	0.87	0.36, 1.77	0.7	1.14	0.76, 1.65	0.5	0.66	0.23, 1.49	0.4

*Determined by generalized linear model for binomial regression. – represents the reference group of each section. N/A, not applicable due to limited/insufficient data and results were omitted from the modeling; OR, odds ratio; SFG, spotted fever group; STG, scrub typhus group; TG, typhus group.

Acute Visit Questionnaire	**Page** 1 of 3 FSS - C -	**Hospital Center Code**	**Patient Number** -

VISIT INFORMATION ☐ **Informed Consent Obtained**

Interview Date | | | | | | | | (dd mm yyyy) **Time** | | | | (24-hour clock)

PATIENT INFORMATION

Name ____________________ (Last) ____________________ (First Name) ____ (MI)

DOB ________/__________/______________ (dd mm yyyy) **Gender** ☐ Male ☐ Female

HISTORY OF PRESENT ILLNESS

Date of First Symptom | | | | | | | | (dd mm yyyy) **Time** | | | | (24-hour clock)

Symptoms, Including First Symptom *(PLEASE CIRLCE)* Duration (days)

	Fever	Y	N	Don't Know	1	2	3	4		>4__________
	Malaise	Y	N	Don't Know	1	2	3	4		>4__________
	Chills	Y	N	Don't Know	1	2	3	4		>4__________
	Muscle Aches	Y	N	Don't Know	1	2	3	4		>4__________
	Rash	Y	N	Don't Know	1	2	3	4		>4__________
					Face	Trunk	Extremities			Other
	Lesion	Y	N	Don't Know	1	2	3	4		>4__________
	# of lesions (PLEASE CIRCLE): 1-5	6-20	>20		Ulcer	Pustule	Nodule			Other
	Joint Pain	Y	N	Don't Know	1	2	3	4		>4__________
	Headache	Y	N	Don't Know	1	2	3	4		>4__________
	Seizures	Y	N	Don't Know	1	2	3	4		>4__________
	Sore Throat	Y	N	Don't Know	1	2	3	4		>4__________
	Cough	Y	N	Don't Know	1	2	3	4		>4__________
	Shortness of Breath	Y	N	Don't Know	1	2	3	4		>4__________
	Nausea	Y	N	Don't Know	1	2	3	4		>4__________
	Vomiting	Y	N	Don't Know	1	2	3	4		>4__________
					Number of episodes last 24h → ___________					
	Abdominal Cramp*	Y	N	Don't Know	1	2	3	4		>4__________
	Diarrhea *	Y	N	Don't' Know	1	2	3	4		>4__________
					Number of episodes last 24h → ___________					
	Bloody Stools *	Y	N	Don't Know	1	2	3	4		>4__________
					Number of episodes last 24h→ ___________					
	Bloody Urine	Y	N	Don't Know	1	2	3	4		>4__________
	Bleeding (e.g. gums)	Y	N	Don't Know	1	2	3	4		>4__________
	Jaundice	Y	N	Don't Know	1	2	3	4		>4__________
	Other (specify):	Y	N	Don't Know	1	2	3	4		>4__________

- **Please collect stool sample for symptoms with * next to them**

1

Acute Visit Questionnaire	Page 2 of 3	Hospital Center Code	Patient Number
	FSS - C -		-

MEDICATIONS

Please mark all medications taken in the last 30 days ☐ Yes ☐ No ☐ Don't Know

☐	Antibiotics	*Specify all*	
☐	Anti-malaria	*Specify all*	
☐	Steroids	*Specify all*	
☐	Chemotherapy	*Specify all*	
☐	Anti-HIV	*Specify all*	
☐	Other	*Specify all*	

TRAVEL HISTORY

Please mark ALL places the patient has traveled in the last two months ☐ Yes ☐ No ☐ Don't Know

Regional	Yes	Specify:____________________
Other province	☐	Specify:____________________
Other country	☐	Specify:____________________

Was the patient in the Jungle in the last two weeks? ☐ Yes ☐ No ☐ Don't Know

EXPOSURE HISTORY

What is the patient's occupation(s)? (Please check the primary occupation only)

☐ Grain Farmer	☐ Poultry Farmer	☐ Cattle/Sheep Farmer	☐ Factory Worker
☐ Fisherman	☐ Office Worker	☐ Driver	☐ Other __________

Does the patient report contact with a person who complained of or showed similar symptoms?

☐ No	☐ Yes	☐ Don't Know
☐ Household member	☐ Neighbor	☐ Other

Does report a history of any the following activities? ☐ Yes ☐ No ☐ Don't Know

IV Drug Use	N Y DK	Alcohol (>2 drinks/d)	N Y DK
Smoking	N Y DK	Unprotected Sex (with some one other than spouse)	N Y DK

PHYSICAL EXAMINATION

Temp _________**ºC** **RR** ___________ (Breaths/minute)

Pulse Rate _________ (Beats/minute)

Blood Pressure (mmHg) ____/______

CLINICAL ASSESSMENT: Diagnosis most consistent with patient's clinical syndrome (pls check ONLY ONE)

☐ Tuberculosis	☐ Pneumonia	☐ Diarrhea
☐ Upper Respiratory Infection	☐ Genital infection	☐ Dysentery
☐ Pharyngitis	☐ Malaria	☐ Other Infection *(specify)*

Acute Visit Questionnaire	Page 3 of 3	Hospital Center Code	Patient Number
	FSS - C -		-

☐ Bronchitis	☐ Typhoid	
☐ Influenza	☐ Dengue	

PATIENT DISPOSITON

☐ Outpatient Follow Up ☐ Admitted to Hospital ☐ Send to Referral Hospital ☐ Patient Dead

FOLLOW UP VISIT INFORMATION ☐ **Patient Refuses or is Not Available**

Date dd mm yy **Time** 24-hour clock

Interviewed by :______________/____________/______________
Name / Signature / Date (dd/ mm/ yy)

Appendix Figure 1. Acute clinical assessment questionnaire template used as part of a cross-sectional prevalence study of patients with acute undifferentiated febrile illness presenting to study site health facilities and tested for rickettsioses as part of a cross-sectional prevalence study in Cambodia from 2007 - 2020.

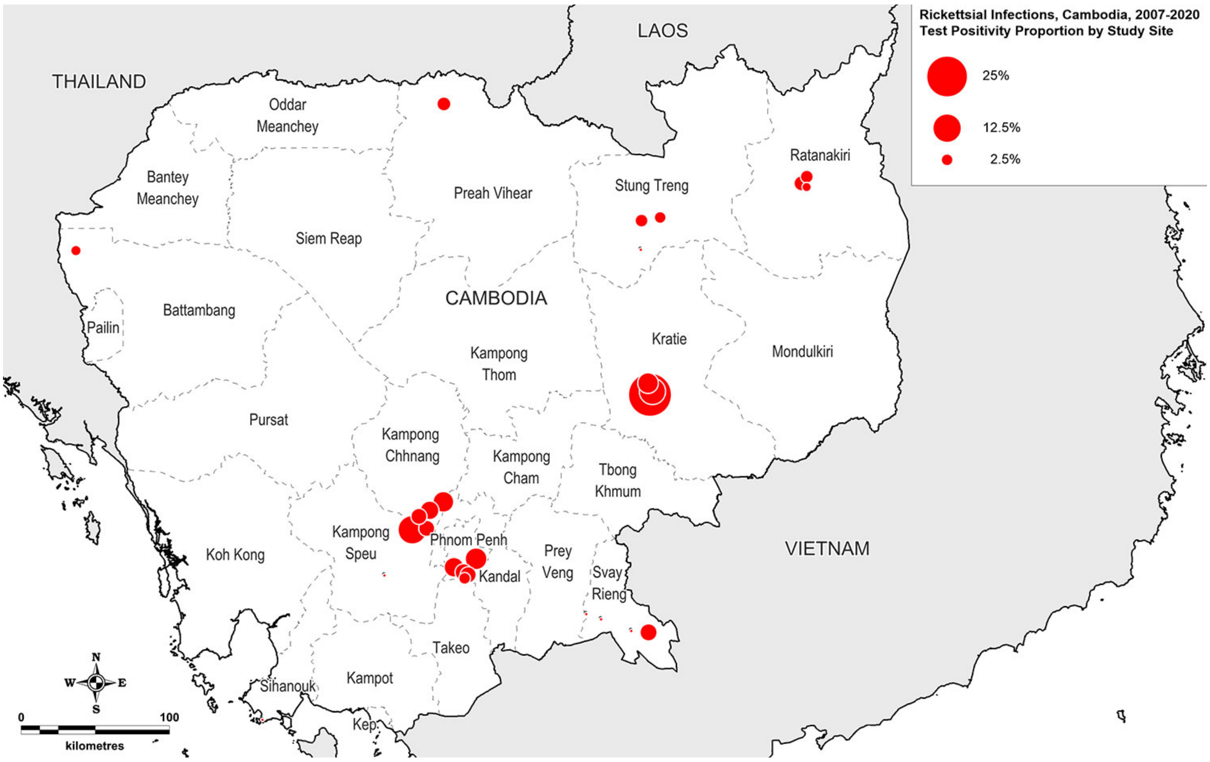

Appendix Figure 2. Test positivity proportions by study site of patients with acute undifferentiated febrile illness presenting to study site health facilities and tested for rickettsioses as part of a cross-sectional prevalence study in Cambodia from 2007 - 2020.
